# Diverse animal models for *Chlamydia* infections: unraveling pathogenesis through the genital and gastrointestinal tracts

**DOI:** 10.3389/fmicb.2024.1386343

**Published:** 2024-03-28

**Authors:** Qi Tian, Tianyuan Zhang, Chuqiang Shu, Zixuan Han, Youyou Huang, Jiao Wan, Luying Wang, Xin Sun

**Affiliations:** ^1^Department of Obstetrics & Gynecology, Hunan Provincial Maternal and Child Health Care Hospital, Changsha, Hunan, China; ^2^National Health Commission Key Laboratory for Birth Defect Research and Prevention, Hunan Provincial Maternal and Child Health Care Hospital, Changsha, Hunan, China; ^3^Key Lab of Molecular Virology and Immunology, Shanghai Institute of Immunity and Infection, Chinese Academy of Sciences, Shanghai, China; ^4^Key Laboratory of Multi-Cell Systems, Shanghai Institute of Biochemistry and Cell Biology, Center for Excellence in Molecular Cell Science, Chinese Academy of Sciences, Shanghai, China; ^5^Department of Obstetrics and Gynecology, 3rd Xiangya Hospital, Central South University, Changsha, Hunan, China

**Keywords:** chlamydia, chlamydia pathogenesis, animal models, genital tract infections, gastrointestinal tract infections

## Abstract

*Chlamydia trachomatis* is responsible for infections in various mucosal tissues, including the eyes, urogenital, respiratory, and gastrointestinal tracts. Chronic infections can result in severe consequences such as blindness, ectopic pregnancy, and infertility. The underlying mechanisms leading to these diseases involve sustained inflammatory responses, yet thorough comprehension of the underlying mechanisms remains elusive. Chlamydial biologists employ in multiple methods, integrating biochemistry, cell biology, and genetic tools to identify bacterial factors crucial for host cell interactions. While numerous animal models exist to study chlamydial pathogenesis and assess vaccine efficacy, selecting appropriate models for biologically and clinically relevant insights remains a challenge. Genital infection models in animals have been pivotal in unraveling host-microbe dynamics, identifying potential chlamydial virulence factors influencing genital pathogenicity. However, the transferability of this knowledge to human pathogenic mechanisms remains uncertain. Many putative virulence factors lack assessment in optimal animal tissue microenvironments, despite the diverse chlamydial infection models available. Given the propensity of genital *Chlamydia* to spread to the gastrointestinal tract, investigations into the pathogenicity and immunological impact of gut *Chlamydia* become imperative. Notably, the gut emerges as a promising site for both chlamydial infection vaccination and pathogenesis. This review elucidates the pathogenesis of *Chlamydia* infections and delineates unique features of prevalent animal model systems. The primary focus of this review is to consolidate and summarize current animal models utilized in *Chlamydia* researches, presenting findings, discussions on their contributions, and suggesting potential directions for further studies.

## 1 Introduction

*Chlamydia*, predominantly represented by *Chlamydia pneumoniae* (C.p) and *Chlamydia trachomatis* (C.t), constitutes a significant portion of human infections. The escalating impact of C.t-mediated diseases emphasizes the urgency for innovative interventions alongside existing public health measures (de la Maza et al., 2017; Gottlieb and Johnston, 2017).

The successful transformation of C.t using recombinant plasmids from its own endogenous plasmid has accelerated the study of plasmid-encoded factors crucial for chlamydial pathogenicity (Ding et al., 2013; Gong et al., 2013; Song et al., 2013). The development of genetic tools has expanded exploration into chromosome-encoded factors, resulting in numerous genetically defined mutants (Brothwell et al., 2018; Putman et al., 2019). The ongoing optimization of these genetic tools calls for a thoughtful discussion on selecting the most suitable models for evaluating these mutants. Despite the availability of various animal models to study chlamydial pathogenic mechanisms and assess vaccine efficacy, refining, and choosing the most appropriate models for obtaining biologically and clinically relevant information remains a challenge.

Our aim is to clarify the general pathogenic characteristics of chlamydial infections and navigate the intricacies of selecting appropriate model systems, recognizing their inherent limitations, to extract biologically and clinically relevant insights. Lastly, we will elaborate on the newly proposed hypothetical model for *Chlamydia* genital-gut interaction to offer a more comprehensive understanding of chlamydial pathogenesis and insights for future research.

We believe in the importance of selecting robust models with appropriate infection routes, coupled with well-matched mutants. This strategic approach enhances our ability to identify tissue-dependent C.t virulence determinants, unravel mechanisms driving site-specific immunity and pathology, and pinpoint site-specific factors that may either contribute to or impede local immunity.

## 2 Genital animal models of *Chlamydia* infection

### 2.1 Non-human primate trachoma and NHP genital tract models

After Tang successfully isolated C.t organisms from human ocular tissues (Tang et al., 1957), attempts were made to infect monkey eyes with human isolates to meet Koch's postulates criteria for trachoma causation (Wang, 1999). Various NHP species exhibited induced ocular inflammatory pathologies similar to those in humans, confirming causation and establishing a valuable NHP ocular model for studying trachoma pathogenesis and vaccine evaluation.

A whole C.t organism-based vaccine induced protective immunity against conjunctivitis in monkeys, but immunity waned within a year. Notably, C.t serovar A, capable of inducing robust inflammatory pathology in monkeys, failed when deficient in the chlamydial plasmid (Kari et al., 2011). The plasmid's role in human ocular infection and pathogenesis needs further investigation, but its correlation with high antibody titers to plasmid-encoded Pgp3 suggests potential contributions to chlamydial pathogenicity in human ocular tissue (Winstanley et al., 2017; Wiegand et al., 2018).

The primate trachoma model revealed persistent live organism shedding from serovar A-infected monkeys' ocular tissue for up to 7 weeks, with inflammatory pathologies lasting up to 15 weeks (Kari et al., 2011). In contrast, plasmid-free serovar A-infected monkeys experienced brief shedding, clearing infection within 3 weeks without significant pathology. This underscores the plasmid's necessity for C.t colonization and pathogenicity in ocular tissue, consistent with its prevalence in human C.t isolates. Moreover, plasmid-free serovar A-inoculated monkeys were protected against both infection and pathology induced by wild-type serovar A, demonstrating the feasibility of developing a live attenuated C.t vaccine (de la Maza et al., 2017; Zhu et al., 2018). Interestingly, ocular protective immunity in macaques was found to be CD8+ T cell-dependent, though its role against C.t infections at other sites remains undetermined (Olivares-Zavaleta et al., 2014).

Over the years, various NHP species, including pigtail and rhesus macaques, have been employed to model C.t genital tract infections (Bell et al., 2011). The genital tracts of female pigtail macaques share many similarities with those of women, including the length of the menstrual cycle, reproductive tract anatomy, cervical tissue cellular structure, and vaginal microflora. In a recent study of C.t pathogenicity in pigtail macaques, six macaques received a cervical inoculation of C.t and were observed and sampled at weekly intervals (Patton et al., 2018). These macaques developed mild to moderate infection and disease. Remarkably, parallel animals inoculated with the same strain of C.t but deficient in the plasmid developed similar infection and disease endpoints (Patton et al., 2018), and these results were reproducible in rhesus macaques (Qu et al., 2015). Evidently, the plasmid is not essential for C.t infection in primate genital tracts, contrasting with its requirement in the ocular C.t challenge model. A baboon cervical infection model exists but may face limitations due to cost and availability issues (Bell et al., 2011).

### 2.2 Mouse model of C.t genital infection

Despite C.t not naturally infecting rodents, mice serve as essential models for exploring chlamydial pathogenic mechanisms (Zhong, 2018) and evaluating vaccine candidates (de la Maza et al., 2017). The efficiency of creating mutations in C.t serovar L2 has led to extensive assessments in mice, including L2 mutants; other C.t organisms, such as serovar D with or without genetic mutations, have also been studied in the murine model (Sturdevant et al., 2010).

However, C.t encounters challenges overcoming murine innate immunity, resulting in its swift elimination from the mouse genital tract (Sturdevant and Caldwell, 2014). In contrast, intravaginal inoculation with *Chlamydia muridarum* (C.m) effectively establishes productive infection and induces upper genital tract pathology, resembling observations in C.t-infected women during laparoscopy (Sun et al., 2015). This robust model, along with genetic manipulation of C.m, has significantly advanced our understanding of chlamydial pathogenesis.

Following Tang's isolation of C.t from human ocular tissues (Tang et al., 1957), mice immunized with killed C.t prevented toxicity from intravenous injection of live C.t, aiding in classifying 'trachoma virus strains' and studying IFNγ's role in chlamydial infection. However, this model lacked relevance to C.t pathogenicity in humans.

To gain more pertinent information on C.t pathogenicity, intravaginal inoculation into mice was deemed necessary. Unfortunately, C.t fails to induce lasting pathology in the mouse upper genital tract, despite lower genital tract infections (Sturdevant et al., 2010; Eko et al., 2014). This is partly due to C.t's inability to overcome innate immunity in the mouse genital tract. Female C3H/HeJ mice, with a natural toll-like receptor 4 gene mutation, show increased susceptibility to C.t in the genital tract (Sturdevant et al., 2010). Improving the mouse intravaginal infection model by genetically increasing susceptibility to C.t colonization is desired.

The intrabursal model, requiring survival surgery and overcoming physiological barriers, contrasts with the preferred transcervical inoculation with C.t due to its less invasiveness. Transcervical inoculation with an inactivated C.t vaccine strain showed protection against live C.t challenge infection (Stary et al., 2015). Additionally, transcervical inoculation with live C.t serovar D induced tubal infertility in C57 wild-type and HLA-DR4 transgenic mice, serving as a potentially useful model for studying C.t pathogenesis and evaluating human-relevant chlamydial vaccines (Pal et al., 2018).

Following the successful transformation of the C.t LGV L2 strain, a series of L2 mutants deficient in plasmid genes were created and assessed in mice (Ramsey et al., 2014). Pgp3-deficient L2 showed attenuation in infecting the mouse lower genital tracts and inducing inflammatory pathology. An L2 strain deficient in expressing the chlamydial protease-like activity factor (CPAF) was isolated from L2 mutagenesis libraries. CPAF-deficient L2 revealed the critical role of CPAF in chlamydial survival in mice. However, the L2 genital infection model has limitations for modeling C.t infection in humans, as both the infection and pathology were transient even with wild-type L2.

### 2.3 Mouse model of genital C.m infection

The mouse cervicovaginal infection model with C.m has been crucial for unraveling chlamydial pathogenic mechanisms and host immune responses (Cheng et al., 2008). Utilizing C.m, chosen for its genomic similarity to C.t, has mirrored upper genital tract pathology observed in women infected with C.t. Evaluation of mouse genital tracts revealed gross pathologies, such as hydrosalpinx (Sun et al., 2015), aligning with medically relevant endpoints seen in C.t-infected women (Budrys et al., 2012).

Upon intravaginal inoculation, C.m ascends the mouse genital tract, triggering acute inflammation manifesting as pyosalpinx detectable macroscopically between 2 and 3 weeks. Some pyosalpinxes transform into hydrosalpinx by the 4^th^ week, potentially leading to long-lasting infertility (Zhang et al., 2014). Long-lasting hydrosalpinx in the C.m mouse model mirrors fibrotic pathologies seen in women with tubal factor infertility (Budrys et al., 2012). The chlamydial plasmid, particularly Pgp3, plays a pivotal role in inducing hydrosalpinx. Deficiency in Pgp3 mirrors plasmid-deficiency effects, emphasizing its major contribution to the overall pathogenic process (Zhong, 2018). And Chromosomal proteins TC0237/TC0668 also contribute to hydrosalpinx induction in the mouse genital tract (Chen et al., 2015; Conrad et al., 2016).

With these models, various mechanisms have been identified that influence chlamydial induction of upper genital tract pathology (Murthy et al., 2011; Frazer et al., 2013). Despite these advancements, our understanding is still incomplete, particularly regarding the mechanisms sustaining the persistence of long-lasting pathogenic tubal fibrosis after *Chlamydia* clearance from the oviduct infection site in animal genital models.

## 3 Gastrointestinal *Chlamydia* infection model

While *Chlamydia* is primarily recognized as a genital tract pathogen, its regular detection in the human gastrointestinal (GI) tracts (Peters et al., 2014; Craig et al., 2015) introduces a layer of complexity. This finding aligns with observations of C.t infecting human enteroendocrine cells (Dlugosz et al., 2014). Remarkably, individuals engaging in oral/anal sex or those abstaining from such behaviors both show C.t presence in their rectal swabs (Peters et al., 2014), suggesting potential routes of C.t transmission from genital to GI tracts beyond sexual behavior.

The mechanism by which C.t enters the GI tract, whether through sexual behaviors or alternative pathways, raises a critical question: can GI tract C.t influence C.t pathogenicity in the genital tract (Bavoil et al., 2017). This inquiry becomes essential due to the frequent detection of C.t in the human GI tract. Addressing this question in human subjects would necessitate extensive clinical investigations, potentially involving therapeutic interventions. As an alternative approach, evaluating the impact of GI tract *Chlamydia* on genital tract pathogenicity can be explored using the murine model of CM induction of hydrosalpinx (Tian et al., 2023).

The successful transformation of C.m by has provided a valuable tool for real-time monitoring of live chlamydial infection in mice (Campbell et al., 2014). This tool has revealed the swift ascent of vaginal C.m to the oviduct, its subsequent spread to the GI tract, and its ability to establish long-lasting colonization within the GI tract (Zhang et al., 2015). In the context of mice, chlamydial long-lasting colonization in the gut denotes the enduring presence and survival of *Chlamydia* within the GI tract over an extended period. While a clear definition in human hosts is currently lacking, the persistence of *Chlamydia* in the large intestine of mice can extend for hundreds of days (>50 weeks), significantly surpassing the duration observed in the genital tract. Chlamydial colonization in the GI tract depends on its overcoming mucosal barriers, utilizing chromosomal genes to evade IFNγ from innate lymphoid cells (Zhong, 2021). Remarkably, the spread from the genital tract to the GI tract appears independent of the oral/anorectal route, as evidenced by experiments with mice wearing restraining Elizabethan collars and housed in net-bottom cages to prevent coprophagy. Instead, this spreading seems to be dependent on multiple pathways (Zhang et al., 2015). The research indicates potential pathways for hematogenous *Chlamydia* to reach the large intestine lumen after genital infection dissemination, involving both spleen-to-stomach and liver-to-intestine routes (Zhou et al., 2021). Additionally, other studies suggest that genital *Chlamydia* can also be transported to the gut by host cells (Howe et al., 2019). Upon arrival in the GI tract, C.m does not autoinoculate extra-GI tissues (Wang et al., 2016), challenging the hypothesis that chlamydial organisms in the GI tract could act as a reservoir for auto-inoculating the genital tract (Yeruva et al., 2013). Despite the absence of autoinoculation, the correlation between chlamydial spreading from the genital to GI tracts and pathogenicity in the upper genital tract has led to the proposition of a Two-Hit model as a chlamydial pathogenic mechanism (Tian et al., 2020).

**Figure 1:**
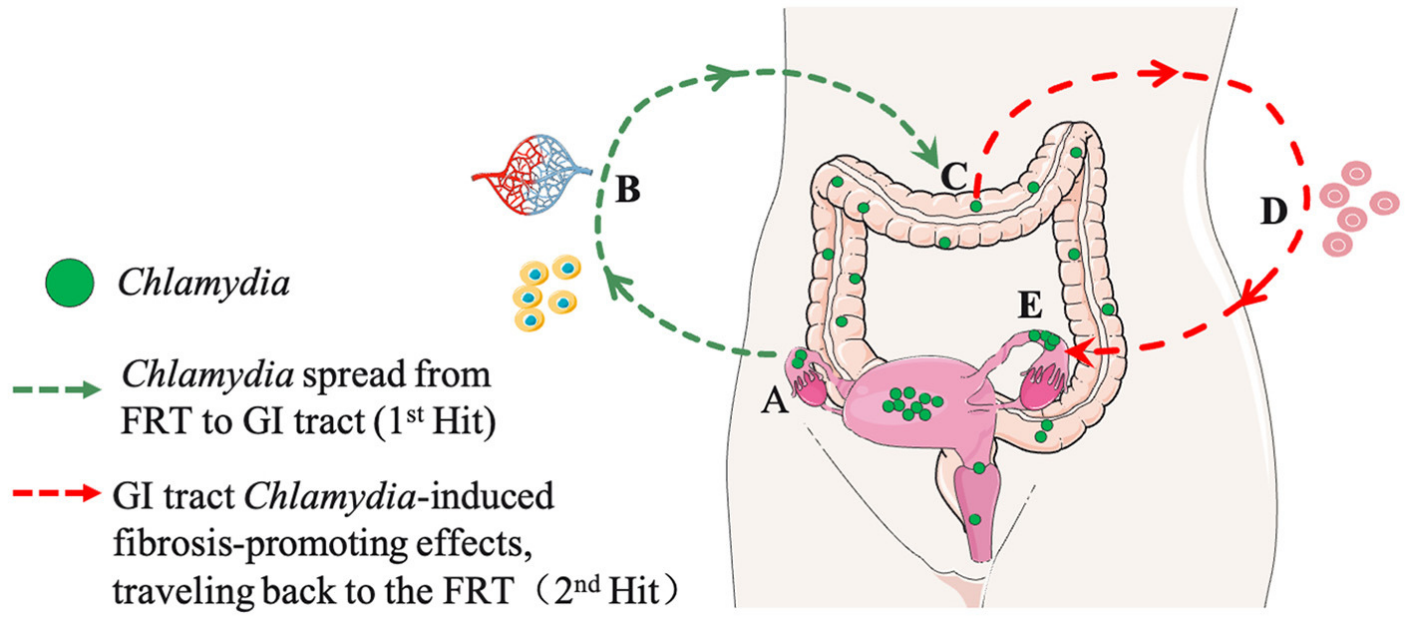
A hypothetical model for *Chlamydia* pathogenesis: interactions between the genital and gastrointestinal (GI) tracts (A Two-Hit Model). **(A)** First Hit: *Chlamydia* ascends the female reproductive tract (FRT), invading oviduct epithelial cells, inducing acute inflammation, and resulting in the clearance of tubal infection, epithelial damage, and transient fibrotic responses. **(B)** Spreading Routes: *Chlamydia* spreads from the FRT to the GI tract through multiple pathways, including blood circulation and host cells. **(C)** GI Tract Colonization: *Chlamydia* further spreads and colonizes the large intestine. **(D)** Second Hit: The colonized gut act as a reservoir for continuous exposure, perpetuating its impact on FRT pathogenicity. This contributes to sustained inflammation, pro-fibrotic responses, and effects on the FRT. **(E)** Long-Term Consequences: The enduring impact from GI tract on tubal fibrosis in the FRT may contribute to infertility and other reproductive complications. Portions of the figure utilized images from Servier Medical Art, licensed under Creative Commons Attribution 3.0 Unported License.

In this Two-Hit model (Figure 1), the first hit is attributed to genital C.m ascending infection, where C.m invades oviduct epithelial cells and induces tubal inflammation. This initial hit may cause epithelial damage, triggering tissue repair responses, including fibrosis. While the fibrotic response is typically transient and halts upon the restoration of oviduct function, the second hit comes into play when genital C.m spreads to the GI tract. Here, C.m organisms residing in the GI tract for extended periods induce pro-fibrotic lymphocytes, which, when recruited into the oviduct previously affected by C.m ascending infection, act as the second hit. And the nature of the second hit might be GI related lymphocytes, such as CD8+T cells (Tian et al., 2021). These GI tract-derived C.m-specific profibrotic lymphocytes may convert the initially transient tubal repairing fibrotic response into a long-lasting tubal fibrotic blockade (Tian et al., 2020). The 2-hit model aligns with the concept that responses induced by gut bacteria can impact tissues beyond the GI tract. Conversely, if a naive mouse is exposed to C.m in the GI tract first, it essentially becomes immune to subsequent C.m exposure in extra-GI tissues (Wang et al., 2018; Zhu et al., 2018). The site of the first exposure to C.m may therefore determine the consequential outcome. It is essential to note that the contribution of gut *Chlamydia* to upper genital pathology has only been tested in mice under specific experimental conditions. Thus, the Two-Hit Model remains a hypothesis requiring further exploration in human studies.

While C.t is frequently detected in the human GI tracts, the impact of human GI tract C.t on C.t pathogenicity in the genital tract remains unclear. Exploring the current studies in human contexts could provide valuable insights into this aspect.

## 4 Discussion

The review emphasizes challenges in modeling chlamydial infections and the limitations of current animal models (Table 1). Host-specific adaptation poses a critical challenge, hindering lasting fibrosis induction in murine genital tracts by genitourinary C.t serovars. While IFNγ-mediated immunity is crucial for both human C.t and murine C.m infections, distinct mechanisms highlight complex host-pathogen interactions.

**Table 1:** Comparative summary of *Chlamydia* animal models.

**Model**	**Infection site**	**Key findings**	**Significance**	**References**
NHP ocular model	Eyes	- Successful isolation of C.t organisms - Induction of ocular inflammatory pathologies similar to humans - Recognizing the role of plasmid-encoded protein Pgp3 in chlamydial pathogenicity - Evaluation of trachoma vaccines	Initiating studies on chlamydia pathogenesis and gaining insights into C.t ocular infection mechanisms, thereby informing strategies for trachoma prevention.	(Tang et al., 1957; Wang, 1999; Kari et al., 2011; Olivares-Zavaleta et al., 2014; de la Maza et al., 2017; Winstanley et al., 2017; Wiegand et al., 2018; Zhu et al., 2018)
NHP genital model	Genital tract	- Evaluation of C.t infection and disease in female macaques - Importance of the chlamydial plasmid - Exploration of vaccine strategies	Unraveling C.t pathogenesis in the genital tract of NHP, and exploration of therapeutic strategies	(Bell et al., 2011; Qu et al., 2015; Patton et al., 2018; Zhu et al., 2018)
Mouse genital model	Genital tract	- Use of C.t/C.mfor productive infection and upper genital tract pathology - Examination of genetic manipulation for understanding *Chlamydia* pathogenesis - Exploration of vaccine strategies	Identifying key factors in chlamydial pathogenicity	(Sturdevant et al., 2010; Eko et al., 2014; Ramsey et al., 2014; Sturdevant and Caldwell, 2014; Zhang et al., 2014; Stary et al., 2015; Sun et al., 2015; Conrad et al., 2016; de la Maza et al., 2017; Pal et al., 2018; Zhong, 2018)
Mouse GI tract model	Gastrointestinal tract	- Detection of C.t in human GI tracts -Discussion of meaning of GI tract Chlamydial infection - Exploration of potential routes of spread from genital to GI tract - Exploration of vaccination through GI tract - Proposal of the Two-Hit model involving both genital and GI tracts in *Chlamydia* pathogenesis	Exploring the GI tract as a novel site for *Chlamydia* infection, discussing potential routes of C.m spread from the genital to the GI tract, and proposing the possible contribution of GI Chlamydia to upper genital tract pathogenesis.	(Yeruva et al., 2013; Campbell et al., 2014; Dlugosz et al., 2014; Craig et al., 2015; Zhang et al., 2015; Wang et al., 2016, 2018; Bavoil et al., 2017; Tian et al., 2020, 2021, 2023)

Tissue tropisms of C.t serovars in humans raise questions about factors influencing tissue-specific evolution. Chlamydial spreading patterns within and between hosts, coupled with infection dose and frequency considerations, add complexity to modeling disease outcomes. NHP and rodent models, valuable in certain contexts, cannot precisely replicate human disease, emphasizing the need for further exploration.

The discussion introduces the GI tract perspective, emphasizing *Chlamydia*'s interaction with different anatomical sites. C.m spread from genital to GI tract prompts consideration of GI *Chlamydia*'s impact on genital tract pathogenicity. The Two-Hit model in murine studies provides a framework, but exploring diverse animal models beyond mice is imperative. Examining different species can offer insights into varied responses and consequences, enhancing our understanding of *Chlamydia* pathogenesis.

In conclusion, complexities in modeling *Chlamydia* infections underscore the need for diverse animal models and continued exploration, especially in non-murine species, to capture nuanced host-pathogen interactions in different anatomical sites and broaden our understanding of GI tract *Chlamydia*.

## Author contributions

QT: Funding acquisition, Writing – original draft, Writing – review & editing. TZ: Funding acquisition, Writing – original draft, Writing – review & editing. CS: Writing – review & editing. ZH: Writing – review & editing. YH: Writing – review & editing. JW: Writing – review & editing. LW: Writing – review & editing. XS: Writing – review & editing.
